# Terpene-Based Eutectic Solvent Microdroplets: A Strategy to Combat Antibiotic-Resistant Helicobacter pylori

**DOI:** 10.1021/acs.langmuir.5c01094

**Published:** 2025-06-03

**Authors:** Marek Brzeziński, Magdalena Chmiela, Matias Picchio, Marcelo Calderón, Weronika Gonciarz

**Affiliations:** † Centre of Molecular and Macromolecular Studies, 86897 Polish Academy of Sciences, Sienkiewicza 112, Lodz 90-363, Poland; ‡ Department of Immunology and Infectious Biology, Institute of Microbiology, Biotechnology and Immunology, Faculty of Biology and Environmental Protection, 196812 University of Lodz, Banacha 12/16, Lodz 90-237, Poland; § POLYMAT, Applied Chemistry Department, Faculty of Chemistry, University of the Basque Country UPV/EHU, Paseo Manuel de Lardizábal,3, Donostia-San Sebastián 20018, Spain; ∥ IKERBASQUE, Basque Foundation for Science, Plaza Euskadi 5, Bilbao 48009, Spain; ⊥ Universidad Tecnológica Nacional, Facultad Regional Villa María, Villa María, Córdoba X5900HLR, Argentina; # Consejo Nacional de Investigaciones Científicas y Técnicas CONICET, CABA, Buenos Aires C1425FQB, Argentina

## Abstract

Terpene-based therapeutic eutectic solvents (THEES) represent an innovative class of green solvents with intrinsic antimicrobial properties, offering new potential for sustainable solutions in microbial infection treatment. This study introduces the first application of THEES-based microdroplets targeting Helicobacter pylori (H. pylori), a critical global health concern due to rising antibiotic resistance. Using microfluidic technology, we developed oil-in-water (O/W) emulsion droplets from three distinct THEES systems: menthol/thymol, menthol/lidocaine, and menthol/eucalyptol. These droplets were generated via a flow-focusing glass-capillary microfluidic device, with a 10 wt % poly­(vinyl alcohol) (PVA) aqueous phase and THEES as the organic phase. The presence of surfactant in the system is expected to diminish the integration of bacterial membranes and improve the antimicrobial efficiency of the emulsion droplets compared to THEES alone. Biocompatibility was first evaluated *via* metabolic activity on L929 mouse fibroblasts, followed by *in vitro* testing evaluation against both standard and antibiotic-resistant H. pylori strains. This novel platform THEES-based microdroplets offers a sustainable and potentially transformative strategy for addressing H. pylori infections, providing a breakthrough in combating antibiotic resistance and can potentially prevent the development of resistance if it is used as an alternative to antibiotics.

## Graphical abstract

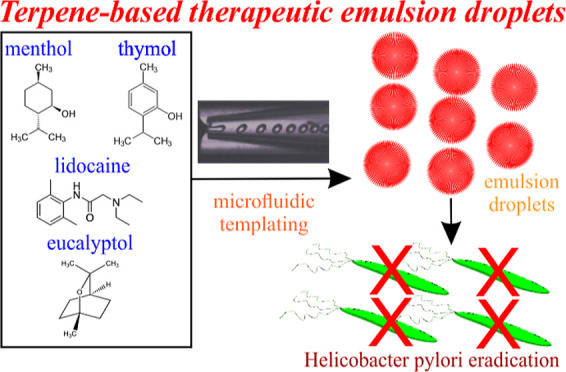

## 1 Introduction

Eutectic solvents are formed by mixing a hydrogen bond donor (HBD) and a hydrogen bond acceptor (HBA), resulting in a liquid mixture with a melting temperature lower than their components.^1,2^ Therapeutic eutectic solvents (THEES) are a class of eutectic systems where at least one component is an active pharmaceutical ingredient (API) or bioactive substance. Some eutectic mixtures have been reported to show substantial negative deviations from the thermodynamic ideality, resulting in an abnormal depression in their melting point.^3^ These eutectic systems are usually called “deep eutectics” as in the archetypical mixture of menthol/thymol. However, currently, there is no consensus on the working definition of deep eutectic solvents, and the term eutectic solvent is preferred to encompass classical and nonideal eutectic systems.

Many THEES formulations have demonstrated antimicrobial properties against various microorganisms, with efficacy depending on their specific composition.^4,5^ Developing emulsion droplets from THEES and incorporating surfactants into the droplet structure can enhance their antimicrobial potency by promoting prolonged and close interaction with bacterial membranes, increasing the likelihood of successful membrane destabilization.^6^ For instance, emulsions containing essential oils exhibit enhanced antimicrobial and antifungal properties compared to their free compounds counterparts due to improved dispersion of emulsion droplets in bacterial and fungal growth media.^7^ Moreover, it was reported that nonionic surfactants in emulsion droplets enhance the hydrophilicity of terpene antimicrobials such as thymol. This increased hydrophilicity promotes stronger interactions with cellular membrane proteins, leading to membrane integrity loss and improved antimicrobial efficacy.^8^

Helicobacter pylori (H. pylori) colonizes the human stomach or duodenum of over 50% of the global population.^9^ It has been shown that H. pylori increases oxidative stress in gastric epithelial cells, which results in the upregulation of apoptosis and gastric barrier dysfunction.^10^ The colonization of gastric mucosa in humans by H. pylori and the local release of soluble components of these bacteria results in the development of chronic inflammatory response, which is correlated with the upregulation of oxidative stress, cell apoptosis, and damage to the gastric epithelial barrier.^11^ This can progress to gastric ulcer or even gastric cancer.^12^ H. pylori has developed various mechanisms to avoid the host immune response. Numerous studies have shown that components of H. pylori inhibit the engulfment capacity of phagocytes, cytotoxic activity, and the expansion of natural killer (NK) cells while also impairing T lymphocyte proliferation.^13^ These strategies help H. pylori to sustain infection. Compounding this, the increasing antibiotic resistance of H. pylori to standard treatments like clarithromycin, amoxicillin, levofloxacin, and metronidazole underscore the urgent need for alternative therapies,^14,15^ including biologically active substances with bactericidal potential. Several studies have revealed that essential oils (EO) containing thymol,^16^ menthol,^17,18^ eugenol,^19^ and limonene^20^ are bactericidal against H. pylori. However, the concentrations of these compounds needed for effective treatment are usually very high and often difficult to maintain at the required levels after oral administration.

Furthermore, eutectic formulations combining different antimicrobial agents could result in synergistic effects. It is also expected that the efficacy and biocompatibility of the synergic combinations are increased by formulating them in emulsions because of the presence of surfactant that may diminish the stability of the biological membrane of bacteria.^5^ Herein, a microfluidic method was employed to prepare the emulsions due to its ability to create monodisperse systems.^21,22^ A flow-focusing geometry was employed for droplet formation, enabling the production of oil-in-water (O/W) emulsions by adjusting the flow rates of the water and oil phases.^23–25^ While traditional methods typically use organic solvents like chloroform, methylene chloride, *n*-hexane, or ethyl acetate,^26,27^ the use of hydrophobic terpene-based THEES as an oily organic phase represents a novel application in microfluidic templating.^28^ In this study, three different THEES, namely, menthol/thymol, menthol/lidocaine, and menthol/eucalyptol, were used as the organic phase, and a 10 wt % solution of poly­(vinyl alcohol) (PVA) in water was employed as the aqueous phase. By adjusting the flow rates, O/W emulsions were prepared. The biocompatibility of these droplets was first assessed using standard L929 mouse fibroblasts to determine their safe concentration range. Subsequently, both the THEES droplets and pure THEES were tested against reference H. pylori and six clinical strains resistant to metronidazole, clarithromycin, or a combination of metronidazole/clarithromycin or metronidazole/levofloxacin.^29^ Our findings demonstrated that the microfluidic process effectively produced THEES-based emulsion droplets with significant antibacterial activity against antibiotic-resistant H. pylori strains. The results obtained in this study prompt further research using the higher number of H. pylori clinical isolates to estimate the effectiveness of the studied formulations’ antibacterial properties and determine their potential antibacterial mechanisms. We hypothesize that the eutectic form of these compounds could significantly enhance their antimicrobial effects against H. pylori due to their higher permeability and solubility in the liquid state, eliminating the need for harmful organic solvents.

## 2 Materials and Methods

### 2.1 Materials

d,l-Menthol–menthol, 2-isopropyl-5-methylcyclo hexanol, C_10_H_20_O (No. W266507, Sigma-Aldrich, Darmstadt, Germany); lidocaine–2-diethylamino-*N*-(2,6 dimethylphenyl), acetamide, C_14_H_22_N_2_O (no. L7757, Sigma-Aldrich); eucalyptol–1,3,3-trimethyl-2oxabicyclo[2.2.2]­octane, 1,8-cineole, 1,8-epoxy-p-methane, C_10_H_18_O (no. 1268900, Merck, Darmstadt, Germany); and thymol–2-isopropyl-5-methylphenol, 2-[(CH_3_)_2_CH]­C_6_H_3_-5-(CH_3_)­OH (no. PHR1134, Sigma-Aldrich). PVA with a molecular weight of 18,000 g mol^–1^ was purchased from Sigma-Aldrich. All cell culture components were from Biowest, Nuaillé, France.

### 2.2 THEES Preparation

The THEES were prepared using the heating method by mixing the two components (HBA and HBD) and heating them at 60 °C under constant stirring until a homogeneous liquid was formed. The selected molar ratios between the components were menthol/thymol (2:1),^30^ menthol/lidocaine (2:1),^31^ and menthol/eucalyptol (1:1).^32^

### 2.3 Preparation of the O/W Emulsion Droplets by Microfluidics, Their Characterization, and Stability

Glass microcapillary microfluidic devices were prepared using standard procedure.^33,34^ Subsequently, those devices prepared O/W emulsions with organic THEES solutions (dispersed phase) and aqueous PVA (continuous phase) solutions. The continuous aqueous phase and the dispersed organic phase were pumped independently at adjustable flow rates using syringe pumps (Harvard Apparatus PhD ultra) connected to the device with polyethylene tubing (Intramedic Clay Adams PE 90, Becton Dickinson, Sparks, MD 21152-0370, USA). An aqueous solution containing 10 wt % of PVA (*M*_n_ = 18.800 g mol^–1^) was used as the continuous phase. As the dispersed phase, the THEES solution comprises menthol-thymol, menthol-lidocaine, or menthol-eucalyptol. The obtained oil-in-water (O/W) emulsions (100 μL) were transferred to a bath filled with 10 mL of 10 wt % of PVA through Teflon tubing. Monodisperse droplets were produced in the controlled dripping regime^35^ using dispersed phase flow rates of 1–10 μL min^–1^, continuous-phase flow rates of 20–100 μL min^–1^, and capillary orifice diameters of 100–400 μm. The stability of emulsion droplets (ED) was tested after 1 month of incubation at room temperature. Moreover, the droplets were placed on the glass plate at room temperature to induce water evaporation during 1 day to observe structure fluctuations. All tests were visualized by optical microscope AE31E (Motic) equipped with Moticam 1080. The emulsion droplets’ size was determined using a Micro-Tec MS21 glass calibration slide and ImageJ software.

### 2.4 Cytocompatibility Assay

*In vitro* cell culture studies were performed with L929 mouse fibroblasts (LGC Standards, Middlesex, UK) to investigate their viability upon exposure to THEES or their emulsion droplets. The cell viability was assessed colorimetrically according to the ISO norm 10993–5 (International Organization for Standardization, 2009; Biological evaluation of medical devices—part 5: tests for *in vitro* cytotoxicity), based on the 3-(4,5-dimethylthiazol-2-yl)-2,5-diphenyltetrazolium bromide (MTT) reduction by the viable cells, as previously described.^36^ The cells (2 × 10^5^ cells/mL were cultured at 37 °C in a 5% CO_2_ in Roswell Park Memorial Institute (RPMI)-1640 medium supplemented with 10% heat-inactivated fetal bovine serum (FBS) and standard antibiotics: penicillin (100 U/mL) and streptomycin (100 μg/mL) (all cell culture components were from Biowest, Nuaillé, France) in the presence or absence of studied formulations, THEES or THESS derived emulsions (100–900 μL/mL). The entire experiment was conducted in a total volume of 100 μL. Reduced MTT crystals by the viable cells were then dissolved in acidic ethanol, and the color reaction was measured spectrophotometrically. The color intensity of dissolved formazan crystals correlates with the metabolic activity of the fibroblasts. Without the tested materials, the cell cultures in the medium alone were used as a positive control (PC) −100% cell viability. In contrast, cells treated with 0.03% H_2_O_2_ were used as a negative control (NC), i.e., 100% dead cells due to cell lysis. The absorbance was measured spectrophotometrically using a Multiskan^EX^ plate reader (Thermo Scientific, Waltham, MA, USA) at 570 nm. MTT reduction relative to untreated cells (%) = (absorbance of treated cells/absorbance of untreated cells × 100 %) × 100 %. The MTT reduction test mentioned above was performed in three independent experiments.

### 2.5 Assessment of Antimicrobial Activity

The antimicrobial efficiency of THEES and resulting emulsion droplets was performed against three reference H. pylori strains: ATTC (American Tissue Type Collection) 700392, CCUG (Culture Collection University of Gothenburg, Sweden) 17874 and 700392, and six clinical H. pylori strains: 1 - (susceptible to metronidazole, levofloxacin and clarithromycin), 2—(resistant to metronidazole), 3—(resistant to metronidazole and levofloxacin), 4 - (resistant to clarithromycin and metronidazole), 5 - (resistant to clarithromycin) and 6 - (resistant to clarithromycin) from the collection of clinical strains, Medical University of Wrocław, Poland, as previously described using the broth microdilution assay according to The European Committee on Antimicrobial Susceptibility (EUCAST) recommendations for testing antibiotic resistance/sensitivity of H. pylori isolates ((https://www.eucast.org/clinical_breakpoints).^37^ H. pylori strains (reference and clinical isolates) were stored at −80 °C in Tris-buffered saline (TBS) containing 20% glycerol and 10% heat-inactivated fetal calf serum (FCS). Bacteria were cultured for 5 days on modified Helicobacter agar (Becton Dickinson, Heidelberg, Germany) under microaerophilic conditions (Gas Pak, Becton Dickinson, Heidelberg, Germany) at 37 °C. Bacteria were then passaged on Helicobacter agar, 3 times every 48 h, as described above. Gram-negative rods were identified each time using the Gram staining method and by assessment of urease and oxidase activity.

To determine antimicrobial activity, THEES or ED solutions were added to a 96-well plate at 50, 40, 30, 20, or 10 μL). Bacteria were harvested by scraping from agar plates, suspended in Brucella broth containing 10% FCS and Skirrow supplement and antibiotics: polymyxin B—0.2 mg, vancomycin—5 mg, trimethoprim—2.5 mg (Becton-Dickinson GmbH, Heidelberg, Germany), pelleted by centrifugation (2000*g*, for 15 min), and then washed twice under the same conditions. Then, the bacterial pellet was suspended in the Brucella Broth with 10% FCS, and the inoculum was adjusted to a 0.5 McFarland scale (1.5 × 10^9^ CFU/mL). Subsequently, bacterial suspension (10 μL) was added to Eppendorf tubes and centrifuged as above. The bacterial pellet was suspended in appropriate volumes (50, 60, 70, 80, and 90 μL) of Brucella Broth containing 10% FCS and added to previously prepared THEES or ED solutions. Control wells containing bacterial culture alone (positive control of bacterial growth), wells with bacterial medium alone (negative control), and wells with reference antibiotics (clarithromycin, amoxicillin, metronidazole) were included. Plates were incubated for 72 h under microaerophilic conditions at 37 °C, and microbial growth was evaluated spectrophotometrically at 595 nm using a SpectraMax i3× MultiMode Microplate Reader (Molecular Devices, San Jose, CA, USA).

The antimicrobial activity of tested formulations was evaluated based on their minimal inhibitory concentration (MIC) or minimal bactericidal concentration (MBC), expressed as the tested THEES or emulsion in μL/ml. The entire experiment was conducted in a total volume of 100 μL. The tests mentioned above were performed in three independent experiments.

The lowest concentration resulting in total growth inhibition of the tested H. pylori strain was taken as MIC. To determine MBC, 10 μL of the culture was collected from each well, where no visible growth of microorganisms was recorded. The culture was plated onto the surface of Helicobacter agar and incubated for 72 h at 37 °C under microaerophilic conditions.

### 2.6 Statistical Analyses

Graphs were prepared using GraphPad Prism 10.0 software (https://www.graphpad.com/, GraphPad Software Inc., San Diego, CA, USA). Data were expressed as median values ± range. The differences between groups were tested using the nonparametric U Mann–Whitney test. The Statistica 13 PL software (https://statistica.software.informer.c.m/13.3software, Kraków, Poland) was used for statistical analysis. Results were considered statistically significant when *p* < 0.05). We used the Shapiro–Wilk test (S–W) to assess normality distribution.

## 3 Results and Discussion

### 3.1 Preparation and Characterization of THEES Microdroplets

Emulsions as drug delivery systems (DDS) are typically used to deliver either hydrophobic or hydrophilic therapeutic compounds, as these systems are composed of water and oil phases in which such active substances are soluble.^38,39^ Emulsion-based DDS can be particularly effective for the oral delivery of various drugs, as encapsulation improves their bioaccessibility and bioavailability.^40^ Water-in-oil nanoemulsions loaded with amoxicillin have been used to eradicate H. pylori.^41^ The prepared DDS demonstrated adequate H. pylori clearance in a mouse model. However, due to the growing antibiotic resistance of H. pylori, novel approaches should be explored.^15^ In this context, emulsions based on THEES present a promising alternative to existing antibiotic-loaded systems. Moreover, the composition and form of THEES or their emulsion droplets are anticipated to determine the antimicrobial properties. Therefore, the conditions for the THEES-based emulsion droplets by microfluidic templating should be developed due to intense trapping energy.^42^ To achieve this, menthol/thymol, menthol/lidocaine, and menthol/eucalyptol were used as the dispersed phase in a microfluidic setup (Figure 1). Various concentrations of PVA (from 2 to 10 wt %) were tested; however, the stability of the emulsion was the highest for 10 wt % of this surfactant. The formation of droplets correlates with the interplay between the deformation of the o/w interface induced by viscous shear and the interface’s resistance to deformation due to surface tension.^43^ The flow rates of organic phase *Q*_0_ and water phase *Q*_w_ were adjusted to achieve the dripping regime in which the ratio of *Q*_0_/*Q*_w_ determines the size of the emulsion droplet.^44^ Uniform droplets were obtained using dispersed-phase flow rates ranging from 1 to 10 μL/min and continuous-phase flow rates between 20 and 100 μL/min, as shown in Movie S1. After formation, the droplets were collected in a vial containing 10 mL of 10 wt % PVA and immediately sealed for storage.

**Figure 1:**
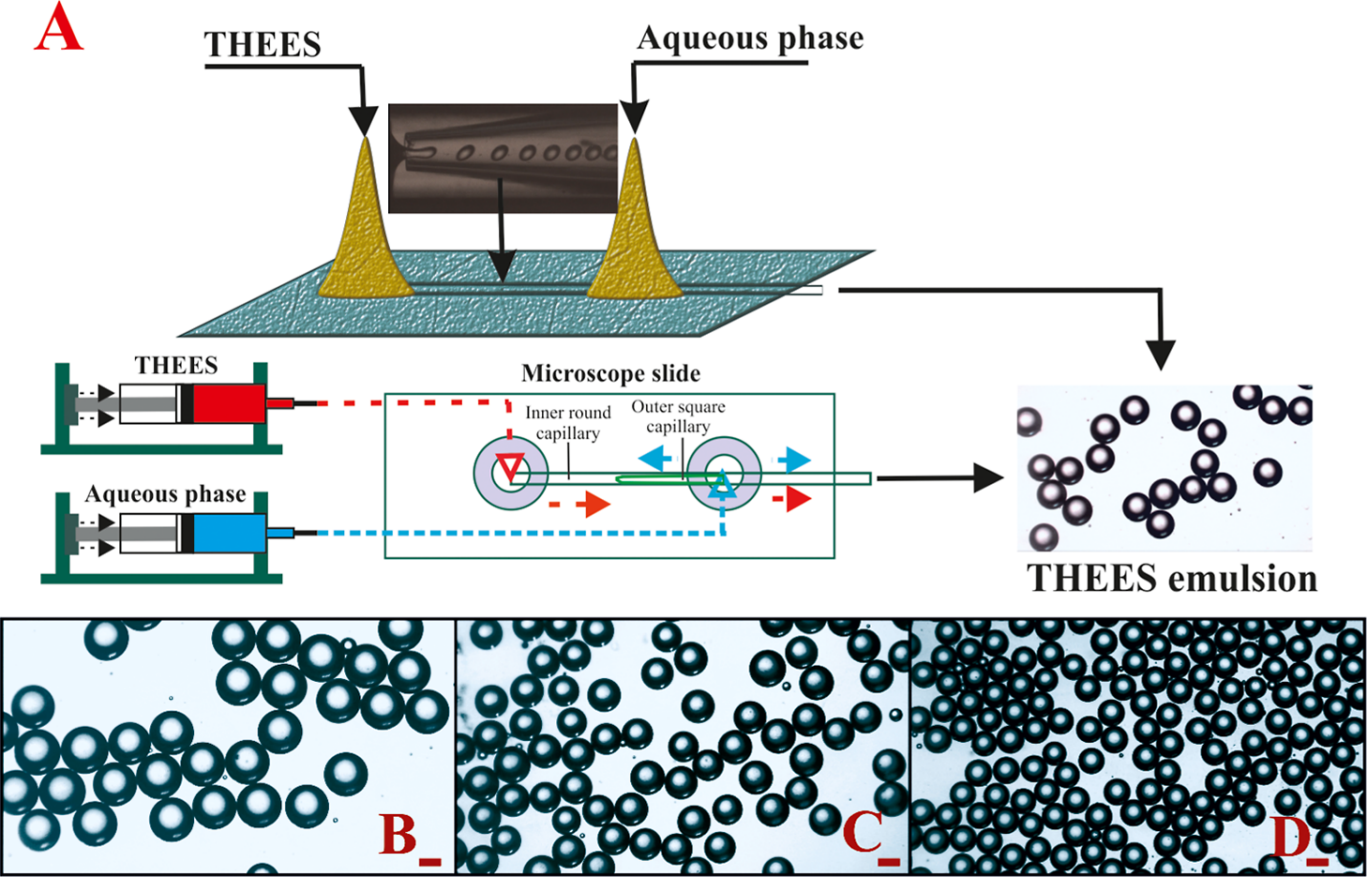
Schematic illustration of the microfluidic device for preparing oil-in-water (O/W) emulsion based on therapeutic eutectic solvents (A). Optical microscopy images of emulsion composed of menthol/thymol (B), menthol/lidocaine (C), and menthol/eucalyptol (D) using the light microscope. The scale bar denotes 100 μm.

The size of the resulting uniform emulsion droplets ranged between 102 and 185 μm, depending on the THEES used, as summarized in Table 1. The largest droplets were produced from menthol/thymol, while the smallest were observed with menthol/eucalyptol. This is correlated with the difference in the viscosity of THEES,^45,46^ and the higher viscosity leads to a bigger emulsion droplet.^47^ The size of these emulsions is suitable for the intended application, as Kwon et al.^48^ demonstrated that droplet size, from millimeter to nanometer scale, does not influence the antimicrobial efficacy of the emulsion; instead, the ingredients are primarily responsible for the biological activity.

**Table 1:** Sizes of the Obtained Emulsion Droplets Determined by an Optical Microscope

type of THEES	min. size [μm]	max. size [μm]	mean [μm]	std. dev. [μm]
menthol/thymol	160	185	172	5.9
menthol/lidocaine	147	181	162	6.7
menthol/eucalyptol	102	143	121	5.9

An essential factor in the stability of the resulting emulsion droplets is the balance between attractive (van der Waals and hydrophobic) and repulsive (electrostatic and steric) interactions.^21^ The obtained emulsion droplets exhibit creaming^49^ due to the difference in density between the obtained droplets and the PVA solution. However, this phenomenon does not affect the overall stability of all obtained emulsion droplets. To support this claim, the storage stability of the example O/W emulsions based on menthol/eucalyptol is shown in Figure 2A­(1,2). The image indicates that the emulsion droplets exhibited no significant changes in size after 1 or 30 days of storage, confirming their adequate storage stability^50^ in sealed vials at room temperature. An investigation of evaporation-induced phase changes in drying oil-in-water emulsion droplets on a glass surface at room temperature was also performed. After 30 min of evaporation, the emulsion droplets retained their shape and size. However, after 1 day, significant changes were observed as shell buckling occurred.^51^ Additionally, the droplets darkened, indicating that evaporation caused the coalescence of the dispersed THEES droplets, and lower light scattering was observed,^52^ as shown in Figure 2B­(1–3). The final stage, characterized by shell formation and buckling, suggests that a layer of agglomerated surfactant covered the remaining mass of the droplets. This layer halts further mass transfer from the droplets, confirming the formation of a solid-like structure.^53^ It could be anticipated that alternatively obtained droplets can cover glass to form an antimicrobial surface.^54^

**Figure 2:**
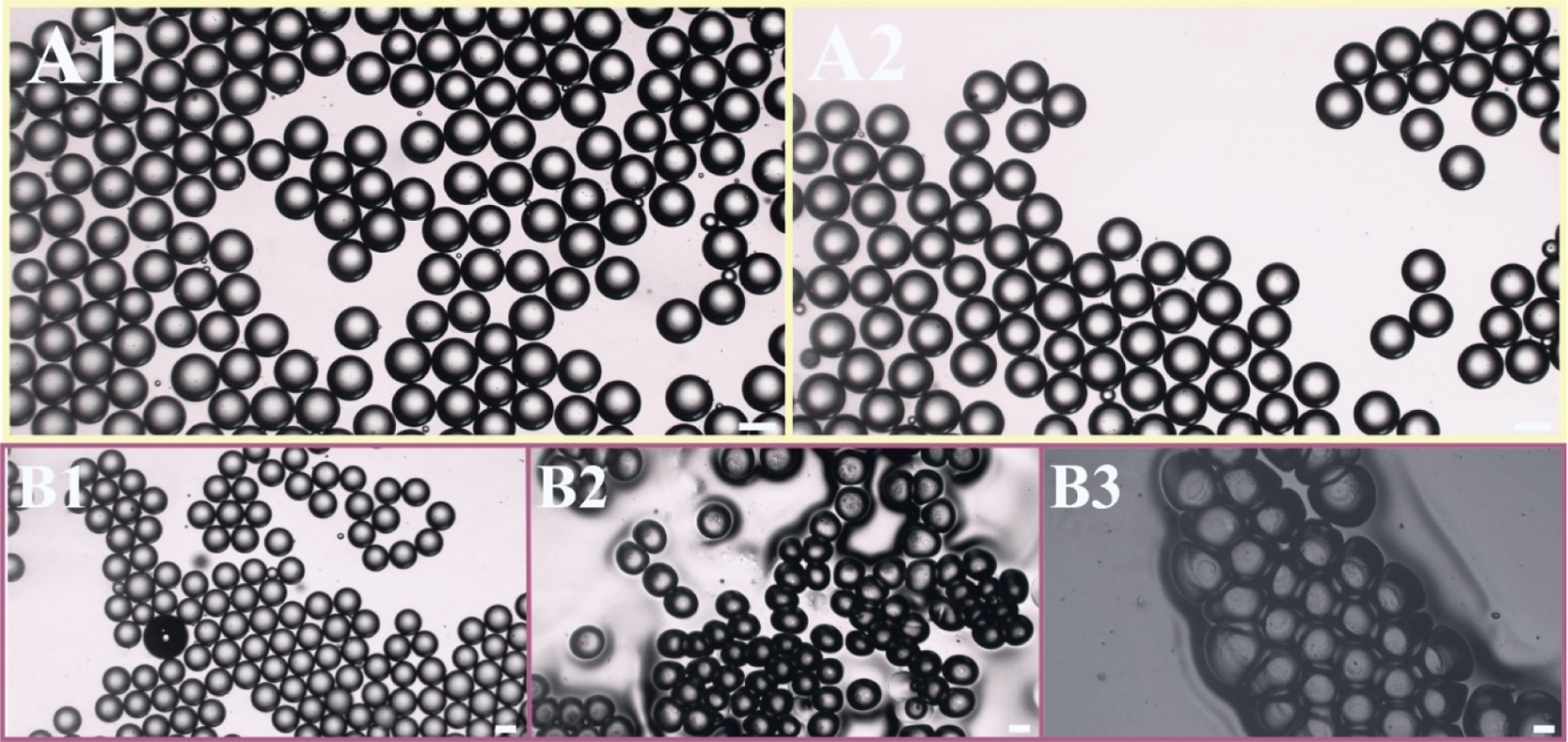
Optical microscopy images of emulsion droplets after 1 day (A1) and 30 days (A2) of storage at room temperature. Drying process of emulsion droplets in room temperature on a glass surface after 30 min (B1), 1 day (B2), and 1 week (B3). An optical microscope was used. The scale bar denotes 100 μm.

### 3.2 *In Vitro* Cytotoxicity and Antimicrobial Properties of THEES and THESS-Derived Microdroplets

The safe concentrations of THEES or THEES-derived emulsion droplets were determined by cell viability assay using L929 mouse fibroblasts, following the ISO standard 10993–5 guidelines. The results revealed cell viability exceeded the 70% safe threshold depending on the THEES composition and concentration. For the liquid THESS, the highest cell viability was observed with menthol/thymol THEES, with a safe concentration of up to 500 μL/mL. In contrast, menthol/eucalyptol eutectic solvent biosafety was limited to concentrations below 300 μL/mL, while menthol/lidocaine THEES solution was safe below 200 μL/mL, as illustrated in Figure 3. Menthol, a known component in the THEES, may contribute to cytotoxicity,^17^ though the second component of the eutectic mixture may also influence THEES toxicity.^55^ In our study, we used the following molar ratios for the mixture components: menthol/thymol (2:1), menthol/eucalyptol (1:1), and menthol/lidocaine (2:1). These compositions have previously been reported to remain liquid over a broad temperature range. The cytotoxicity results suggest that the presence of lidocaine in the menthol/lidocaine THEES may be responsible for its higher cytotoxicity compared to the menthol/thymol THEES.

**Figure 3:**
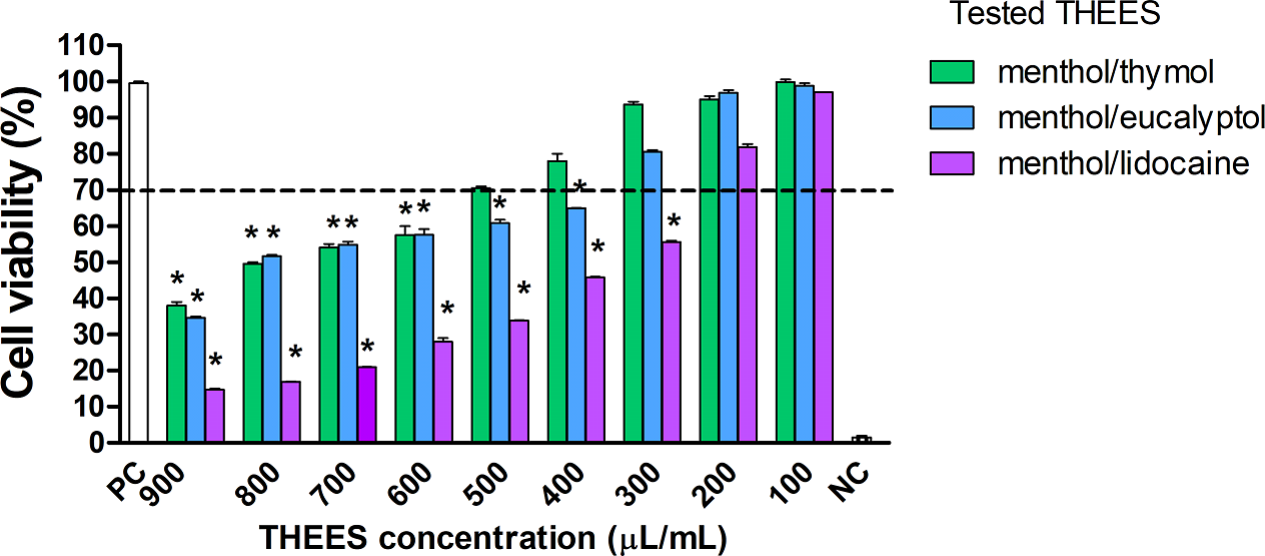
Influence of all tested THEES on cell viability in MTT reduction assay. The cell viability was estimated as the percent of cells that were able to reduce tetrazolium salt (3-(4,5-dimethylthiazol-2-yl)-2,5-diphenyltetrazolium Bromide) (MTT). NC—negative control (cells treated with 0.03% H_2_O_2_), PC-positive control (cells in medium alone, 100% cell viability). Results are shown as median values ± range, *n* = 3. The black line indicates the minimal percentage of viable cells (70%) required to confirm the biomaterial as noncytotoxic *in vitro*. Statistical analysis was performed using the nonparametric U Mann–Whitney test with significance, *p* < 0.05 (*unstimulated cells vs stimulated cells).

Different mechanisms of THEES toxicity toward eukaryotic cells have been suggested.^56^ One hypothesis is that THEES can lower intracellular pH and induce the formation of reactive oxygen species (ROS), which are cytotoxic to human and animal cells. It has been shown that lidocaine induces caspase-dependent apoptosis of rabbit corneal endothelial cells in a time- and dose-dependent manner on the mitochondrial pathway.^57^ Electrostatic interactions between the HBD and HBA also play a role in the formation of THEES and their toxicity.^58,59^ The addition of water may cause disruption of their structure and the interactions with functional groups at the cells’ surface may also play an important role.^60^

Natural antimicrobials often require sufficient concentration to achieve the antibacterial effect. Studies by Xu et al.^61^ and Pan et al.^62^ revealed that the antimicrobial activity of thymol is related to the depolarization of bacteria’s cell plasma membrane. However, a high concentration of thymol is required to acquire this effect.

Various THEES have been tested against multiple bacterial strains, showing great potential.^4,5,63,64^ However, these studies are mainly based on hydrophilic DES from organic acids. Studies with hydrophobic terpenes are limited. Recently, it has been reported that natural antimicrobials such as menthol can be used as high-concentration components to prepare hydrophobic eutectic solvents.^65,66^ The current study has been dedicated to testing the antimicrobial activity of THEES and THEES-derived emulsion droplets against H. pylori reference strains and antibiotic-resistant strains. In 2017, the WHO recommended searching for new antibacterial substances to eradicate H. pylori clarithromycin-resistant strains.^29^

The MIC and MBC of all THEES and their corresponding emulsions were tested against two H. pylori reference strains and several clinical strains resistant to standard antibacterial drugs. As shown in Table 2, it was found that both MIC and MBC of all pure THEES against the H. pylori reference strains and the clinical strain H. pylori 1 sensitive to selected antibiotics (amoxicillin, levofloxacin or metronidazole) were below 100 μL/mL of the THEES, independently of THEES variant. Interestingly, all tested THEES were noncytotoxic to eukaryotic cells in this concentration (Figure 3). Regarding clinical H. pylori isolates, only for H. pylori 5 and H. pylori 6 (both resistant to clarithromycin), MIC or MBC of menthol/eucalyptol were below 100 μL/mL. Similarly, the MIC of this eutectic mixture was 100 μL/mL toward H. pylori 3 (resistant to metronidazole and levofloxacin). For strain H. pylori 2 (resistant to metronidazole or clarithromycin and metronidazole), MIC or MBC of tested eutectic mixtures were in the range of 400–500 μL. In contrast, for H. pylori 4 with the same antibiotic resistance profile, MIC and MBC for all studied THEES were 500 μL/mL. MIC and MBC concentrations of all studied THEES, which are in the range of 100–200 μL/mL, meet the biosafety criterion, as shown in Figure 3. Considering this criterion, the menthol/eucalyptol THEES appears to be the most promising candidate for an antibacterial formulation against H. pylori. MIC of 100 μL/mL or below was demonstrated for 6 out of 8 *H. pylori-tested* strains. H. pylori strains resistant to metronidazole/clarithromycin (H. pylori 2 and H. pylori 4) seem less sensitive to tested THEES. However, further studies using more antibiotic-resistant or antibiotic-sensitive strains are needed to see whether this relationship is substantial.

**Table 2:** Antimicrobial Activity of Studied THEES^a^

*H*. *pylori* strains	MIC/MBC (THEES μL/mL)						tested drug
	menthol/thymol		menthol/lidocaine		menthol/eucalyptol		
	MBC	MIC	MBC	MIC	MBC	MIC	
	reference strains						
*H. pylori* ATCC 700392	<100	<100	<100	<100	<100	<100	sensitive to amoxicillin, metronidazole, levofloxacin
*H. pylori* CCUC 17874	<100	<100	<100	<100	<100	<100	sensitive to amoxicillin, metronidazole, levofloxacin
	clinical strains						
*H. pylori* 1	<100	<100	<100	<100	<100	<100	sensitive to amoxicillin, metronidazole, levofloxacin
*H. pylori* 2	400	400	400	400	500	400	resistant to metronidazole
*H. pylori* 3	500	300	500	200	300	100	resistant to metronidazole and levofloxacin
*H. pylori* 4	500	500	500	500	500	500	resistant to clarithromycin and metronidazole
*H. pylori* 5	500	500	500	400	<100	<100	resistant to clarithromycin
*H. pylori* 6	500	500	500	500	<100	<100	resistant to clarithromycin

a MIC-minimum inhibitory concentration; MBC-minimum bactericidal concentration.

Due to their low water solubility, the utilization of hydrophobic natural biocomponents with antimicrobial properties is limited. The emulsification procedure may help solve this problem. It has been reported that nanoemulsions facilitate the encapsulation of essential oils in small droplets, which improves their application potential, including antimicrobial properties.^67–69^

Using microfluidic technology, we developed oil-in-water (O/W) emulsion droplets from three distinct THEES systems: menthol/thymol, menthol/lidocaine, and menthol/eucalyptol. Emulsion droplets were derived from the studied THEES as organic phase (100 μL) using the 10 wt % PVA (10 mL) as an aqueous phase. The presence of surfactant in the system is expected to diminish the integration of bacterial membranes and improve the antimicrobial efficiency of the emulsion droplets compared to the liquid THEES. The biocompatibility of tested THEES-derived emulsion droplets and of 10 wt % PVA diluted in a culture medium in the following ratio: 90:10, 80:20, 70:30, 60:40, 50:50, 40:60, 30:70, 20:80, and 10:80 were assessed based on the ability of the reference L929 fibroblasts to reduce MTT. The final concentration of THEES in the emulsion droplets was in the range of 1–9 μL/mL (Figure 3).

The emulsion droplets based on menthol/thymol THEES were safe below 200 μL/mL (2 μL/mL of THEES), menthol/eucalyptol below 300 μL/mL (3 μL/mL of THEES), and menthol/lidocaine at 100 μL/mL (3 μL/mL of THEES). Similarly, as in liquid THEES, lidocaine in menthol/thymol emulsion droplets could be responsible for diminishing the viability of L929 fibroblasts (Figure 4).

**Figure 4:**
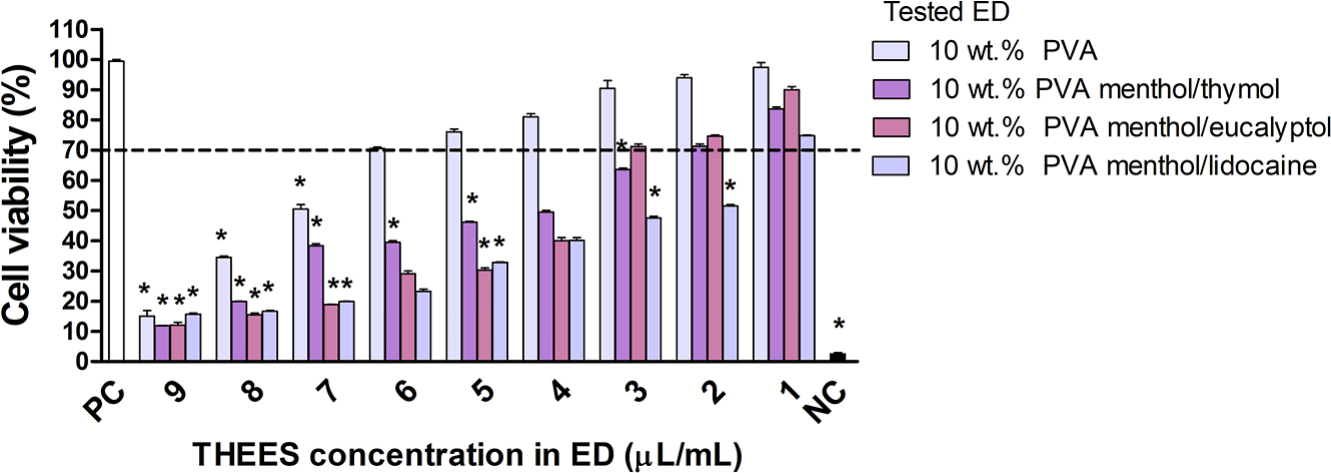
Influence of tested ED on cell viability in MTT reduction assay. The cell viability was estimated as the percent of cells that were able to reduce tetrazolium salt (3-(4,5-dimethylthiazol-2-yl)-2,5-diphenyltetrazolium bromide) (MTT). NC—negative control (cells treated with 0.03% H_2_O_2_), PC-positive control (cells in medium alone, 100% viable cells). Results are shown as median values ± range, n = 3. The black line indicates the minimal percentage of viable cells (70%) required to confirm the biomaterial as noncytotoxic *in vitro*. Statistical analysis was performed using the nonparametric U Mann–Whitney test with significance, *p* < 0.05 (*unstimulated cells vs stimulated cells).

It has been shown that the presence of surfactants in emulsion droplets facilitates their interactions with biological membranes,^6^ which may explain the decrease in cell viability in the presence of THEES-derived emulsion droplets. In this study, 10 wt % PVA was not cytotoxic in the 100–500 μL/mL range. However, this may suggest that coating cells with droplets may facilitate a more substantial influence of droplets’ cargo on cell viability by diminishing the physiological cell functions (Figure 4).

The MIC and MBC concentrations of THEES-derived ED and 10 wt % PVA alone toward H. pylori reference and clinical strains were expressed in μL/ml and are shown in Table 3.

**Table 3:** Antimicrobial Activity of Studied THEES-Derived ED^a^

*Helicobacter pylori* strains		MIC/MBC (μL/mL)							
	10 wt % PVA		ED menthol/thymol 10 wt % PVA vs THEES concentration in ED		ED menthol/lidocaine 10 wt % PVA vs THEES concentration in ED		ED menthol/eucalyptol 10 wt % PVA vs THEES concentration in ED		
	MBC	MIC	MBC	MIC	MBC	MIC	MBC	MIC	
	reference strains								
*H. pylori* ATCC 700392	500	500	<100	<100	<100	<100	<100	<100	sensitive to amoxicillin, metronidazole, levofloxacin
			<1.0	<1.0	<1.0	<1.0	<1.0	<1.0	
*H. pylori* CCUC 17874	500	500	<100	<100	<100	<100	<100	<100	sensitive to amoxicillin, metronidazole, levofloxacin
			<1.0	<1.0	<1.0	<1.0	<1.0	<1.0	
	clinical strains								
*H. pylori* 1	500	500	<100	<100	<100	<100	<100	<100	sensitive to amoxicillin, metronidazole, levofloxacin
			<1.0	<1.0	<1.0	<1.0	<1.0	<1.0	
*H. pylori* 2	500	500	400	300	300	200	100	100	resistant to metronidazole
			4.0	3.0	3.0	2.0	10	1.0	
*H. pylori* 3	500	500	400	400	300	200	100	<100	resistant to metronidazole and levofloxacin
			4.0	3.0	3.0	2.0	1.0	<1.0	
*H. pylori* 4	500	500	500	500	300	300	200	200	resistant to clarithromycin and metronidazole
			5.0	5.0	3.0	3.0	2.0	2.0	
*H. pylori* 5	500	500	500	500	300	200	<100	<100	resistant to clarithromycin
			5.0	5.0	3.0	2.0	<1.0	<1.0	
*H. pylori* 6	500	500	500	300	300	200	<100	<100	resistant to clarithromycin
			5.0	3.0	4.0	2.0	<1.0	<1.0	

a MIC-minimum inhibitory concentration; MBC-minimum bactericidal concentration. The values below reflect the final concentration of THEES in the emulsion droplets.

MIC and MBC for 10 wt % PVA surfactant alone were 500 μL/mL for all tested H. pylori strains. Furthermore, all emulsion droplets derived from THEES demonstrated bactericidal and bacteriostatic effects against reference and clinical H. pylori strains. However, individual strains differed in the sensitivity to the tested emulsion droplets. The H. pylori reference strains and clinical isolate H. pylori 1 were sensitive to all tested emulsion droplets below 100 μL/mL, corresponding to a concentration below 1 μL/mL THEES in the emulsion droplets. MIC and MBC below 100 μL/mL and 1 μL/mL, respectively, were demonstrated for menthol/eucalyptol emulsion droplets against H. pylori strains 2, 3, 5, and 6. Notably, 100 μL/mL of tested emulsion droplets did not induce cytotoxic effects toward eukaryotic cells. MIC and MBC for menthol/thymol and menthol/lidocaine formulations were in the range of 200–500 μL/mL of emulsion droplets, corresponding to 2–5 μL/mL of THEES in the formulation. These preliminary results indicate that, when considering both biosafety criteria and antibacterial activity, the emulsion droplets derived from menthol/eucalyptol eutectic solvent seem to be the most promising formulation for eliminating *H. pylori in vitro*.

However, to elucidate the advantages of the selected formulation, i.e., emulsion droplets derived from menthol/eucalyptol mixture against H. pylori, further *in vitro* studies involving a higher number of well-classified clinical isolates are needed to identify the mechanisms responsible for the observed antimicrobial effect of menthol/eucalyptol emulsion droplets. Although MIC and MBC for the PVA solution alone were 500 μL/mL, the MIC and MBC were much lower for the tested emulsion droplets, suggesting a synergistic effect of both the PVA and the emulsion droplet content. Furthermore, this synergistic effect is also indicated by the final concentration of THEES in studied emulsion droplets, which is 100 times lower than in THEES alone. This shows that potentially different mechanisms dependent on PVA and THEES may determine the intensity of the antibacterial effect of studied emulsion droplets and can be combined with their stability due to the presence of the surfactant. The antimicrobial mechanism of THEES formulations may be attributed to their dehydrating effects on bacterial cells, which can rapidly extract water from the cells, ultimately causing cell lysis.^64^ Furthermore, the components of THEES and the resulting emulsion droplets may increase cell membrane permeability, causing the leakage of intracellular materials.^70^ The improved interaction with biological membranes may also enhance this effect due to the presence of polymeric surfactant. Our results are compatible with this suggestion. Two examples of THEES-based emulsions, one composed of menthol/dodecanoic acid stabilized with partially oxidized cellulose nanoparticles.^71^ Given the current promising research results on the antibacterial activity of menthol/eucalyptol emulsion droplets toward H. pylori, further *in vitro* studies are needed to identify the mechanisms responsible for the observed antimicrobial effect of this formulation involving a higher number of clinical strains. Validating these findings in an experimental infection model in animals sensitive to H. pylori is also necessary. Notably, the Cavia porcellus model we previously characterized offers a convenient option for such studies after os administration of THEES.^37^ The obtained emulsion can be used to fill a gelatin capsule and seal; moreover, E100 can be further employed to cover their surface^72^ and induce colon targeting.

## 4 Conclusions

In this study, we developed novel oil-in-water (O/W) emulsions based on THEES: menthol/thymol, menthol/lidocaine, and menthol/eucalyptol and determined their antimicrobial activity toward sensitive or resistant H. pylori strains. Engineering microfluidic methodologies for producing micrometer-scale emulsions from THEES presented a significant challenge. Motivated by this, we successfully fabricated THEES-based emulsion droplets. Additionally, the emulsions were stabilized with an emulsifier approved by the U.S. Food and Drug Administration (FDA) for pharmaceutical applications, and the presence of PVA ensured appropriate storage stability. Antimicrobial assays demonstrated that both the THEES and their microemulsions exhibited activity against reference and antibiotic-resistant clinical H. pylori strains. The THEES-based microemulsions of menthol/thymol, menthol/lidocaine, and menthol/eucalyptol developed here for treating H. pylori infections have shown antibacterial potential *in vitro*, especially of menthol/eucalyptol emulsion droplets. This formulation was bacteriostatic or bactericidal in a concentration of 100 μL/mL, corresponding to 1.0 μL/mL of THEES cargo, against all tested H. pylori strains, including strains resistant to antibiotics used for the treatment of H. pylori infections. Moreover, this menthol/eucalyptol emulsion droplet concentration was safe for the reference eukaryotic cells. It is anticipated that after *in vivo* oral administration to the gastrointestinal tract, adsorption would occur within 24 h postadministration.^73^ It is promising that this research direction will allow the development of a new formulation for treating H. pylori infection.

## Supplementary Material





## Notes

The formation of uniform droplets of THEES in the microfluidic device (AVI)
(DOCX)
