# Supplementary material for: Terpene-Based Eutectic Solvent Microdroplets: A Strategy to Combat Antibiotic-Resistant Helicobacter pylori
Source: Langmuir. 2025 Jun 3;41(23):14915–24. doi: 10.1021/acs.langmuir.5c01094 (PMC12177948; doi:10.1021/acs.langmuir.5c01094)
Supplement: Supplementary file 2 [file la5c01094_si_002.docx]

**Supporting Information**

Terpene-Based Eutectic Solvent Microdroplets: A Strategy to Combat Antibiotic-Resistant Helicobacter pylori

Marek Brzeziński^1*^, Magdalena Chmiela^2^, Matias Picchio^3,4^, Marcelo Calderón^3,4^, Weronika Gonciarz^2*^

^1^Centre of Molecular and Macromolecular Studies, Polish Academy of Sciences, Sienkiewicza 112, 90-363 Lodz, Poland

^2^Department of Immunology and Infectious Biology, Institute of Microbiology, Biotechnology and Immunology, Faculty of Biology and Environmental Protection, University of Lodz, Banacha 12/16, 90-237 Lodz, Poland

^3^POLYMAT, Applied Chemistry Department, Faculty of Chemistry, University of the Basque Country UPV/EHU, Paseo Manuel de Lardizábal, 3, 20018 Donostia-San Sebastián, Spain

^4^IKERBASQUE, Basque Foundation for Science, Plaza Euskadi 5, 48009 Bilbao, Spain

Corresponding authors’ email: mbrzezin@cbmm.lodz.pl; weronika.gonciarz@biol.uni.lodz.pl

Movie S1. The formation of uniform droplets of THEES in the microfluidic device.
